# Acute Physiological Predictors of Invasive Ventilation and Mortality in Geriatric Intensive Care Unit (ICU) Patients: A Retrospective Cohort Study From India

**DOI:** 10.7759/cureus.110695

**Published:** 2026-06-11

**Authors:** Bhrigu Jain, Aparajit Ballav Dey, Prasun Chatterjee, Avinash Chakrawarty

**Affiliations:** 1 Department of Geriatric Medicine, All India Institute of Medical Sciences, New Delhi, IND; 2 Department of Geriatric Medicine, Artemis Hospital, Gurugram, IND

**Keywords:** geriatric icu, invasive mechanical ventilation, low-resource setting, mortality, physiological predictors

## Abstract

Background

Older adults constitute an increasing proportion of ICU admissions in low- and middle-income countries (LMICs), yet predictors of invasive mechanical ventilation (IMV) and in-hospital mortality remain poorly characterized in dedicated geriatric ICUs. We examined whether acute physiological parameters measured at admission carry greater prognostic value for these two outcomes than baseline comorbidity burden in critically ill older adults.

Methods

We conducted a single-center retrospective cohort study of 291 consecutive patients aged ≥60 years admitted to the dedicated Geriatric Medical ICU at AIIMS New Delhi between June and December 2020. Admission demographics, comorbidities, functional status, and physiological and laboratory parameters were extracted from electronic records. Independent predictors of IMV were identified by multivariable logistic regression and of in-hospital mortality by Cox proportional-hazards modeling. Model discrimination was summarized by the area under the ROC curve (AUC) for IMV and Harrell’s concordance index (C-index) for mortality.

Results

The median age was 76 years (IQR: 69-80); 176 patients (60.5%) were male. IMV was required in 124 patients (42.6%), and in-hospital mortality was 47.1% (137 patients). Independent predictors of IMV were respiratory rate (adjusted OR [aOR] 1.05, 95% CI 1.01-1.10), oxygen saturation (aOR 0.94, 0.91-0.98), Glasgow Coma Scale (aOR 0.84, 0.78-0.92), and PaCO₂ (aOR 1.02, 1.00-1.04). Model AUC 0.73 (0.67-0.79). In the primary mortality model (n=265, which excluded lactate owing to 34% missingness), the independent predictors were diastolic blood pressure (aHR 0.98, 0.97-0.99), bicarbonate (aHR 0.96, 0.93-0.98), and Glasgow Coma Scale (aHR 0.95, 0.91-1.00); age was not independently associated (aHR 1.02, p=0.08). C-index 0.70. In a secondary model restricted to patients with a measured admission lactate (n=186), lactate was independently associated with mortality (aHR 1.22, 1.10-1.36). Baseline multimorbidity was not independently associated with either outcome. Kaplan-Meier analysis demonstrated significantly worse survival among patients aged ≥75 years (log-rank p=0.002) and among those with admission lactate ≥2 mmol/L (log-rank p<0.0001).

Conclusions

In this dedicated geriatric ICU cohort, the degree of acute physiological derangement at admission rather than chronological age or comorbidity burden was the dominant predictor of both invasive mechanical ventilation and in-hospital mortality. Because these parameters are captured routinely at the bedside, they provide a practical basis for early risk stratification and escalation-of-care decisions in resource-limited geriatric critical care.

## Introduction

Population aging is emerging as one of the most significant demographic transitions of the twenty-first century. The global older adult population is projected to roughly double over the next quarter-century, rising from around one billion in 2020 to more than two billion by 2050, with most of this expansion concentrated in low- and middle-income countries [1]. India is experiencing one of the most rapid age structural shifts in the world. The 2023 UNFPA India Ageing Report estimates that older adults (≥60 years) accounted for 10.5% of the population (about 149 million) in 2022 and will reach 20.8% (roughly 347 million) by 2050, growing at a decadal rate of approximately 41% [2]. This demographic shift is accompanied by increasing prevalence of chronic disease, multimorbidity, and frailty, placing substantial pressure on healthcare systems and critical care services.

Aging is associated with progressive decline in physiological reserve across multiple organ systems, which collectively reduces the capacity of older adults to tolerate acute physiological stress [3]. As a consequence, the number of older adults requiring hospitalization and intensive care unit (ICU) admission is rising worldwide. In high-income countries (HICs), older adults now constitute a substantial proportion of ICU admissions. Individuals aged 80 years or older account for approximately 10-20% of ICU admissions in many European and North American centers, with reported hospital mortality ranging from 35% to 55% [4,5]. Similar patterns have emerged in Asian and other low- and middle-income countries (LMICs), though with a higher infectious disease burden.

In India, the burden of older adult patients requiring ICU care is even more amplified by delayed presentation, high infectious disease prevalence, and resource constraints [6]. Observational studies from Indian ICUs have reported in-hospital mortality rates ranging from 14% to 36% among older adults, with sepsis, pneumonia, respiratory failure, and multi-organ dysfunction as the predominant causes of admission and death. Furthermore, recent studies have shown that acute physiological derangement and the evolution of organ dysfunction rather than chronological age or baseline comorbidity burden are the major determinants of short-term outcomes. For example, a 2014 single-center study demonstrated that respiratory acidosis, mechanical ventilation, and inotrope requirements independently predicted mortality in geriatric medical ICU patients, independent of the age of the patients. Similarly, a 2021 prospective study of 100 elderly patients showed that SOFA score at 48 hours and change in SOFA score were excellent predictors of outcome, with mechanical ventilation, inotrope use, renal dysfunction, and impaired consciousness on day two strongly associated with mortality. More recent 2023 data reports that older adults' ICU mortality is 14-22.5%, again highlighting respiratory illness as the leading diagnosis and acute organ support needs as the strongest prognostic factors [7-9]. These findings resonate with multinational cohorts, including the Very Old Intensive Care Patients (VIP) studies in Europe and international modeling efforts such as ELDER-ICU, which also demonstrated that frailty, early organ dysfunction, and acute physiological markers are far stronger determinants of mortality than age alone among patients aged ≥80 years [10-12].

However, most Indian studies remain single-center, derive from mixed-age ICUs, or lack multivariable analyses that separate acute physiological derangement from baseline comorbidity burden. Distinguishing these factors is particularly important in resource-limited settings, where simple admission parameters can guide timely escalation or de-escalation decisions. These gaps underscore the need for dedicated geriatric medical ICU research that moves beyond chronological age to identify robust, multidimensional measures of vulnerability and illness severity in critically ill older adults. The specialized geriatric medical ICU at the Department of Geriatric Medicine, AIIMS New Delhi, offers a unique opportunity to address this gap by studying homogeneous cohorts of older adults with complex medical illness. In this retrospective cohort study, we evaluated the clinical characteristics and outcomes of patients aged ≥60 years admitted to this unit. Specifically, we identified independent predictors of invasive mechanical ventilation and in-hospital mortality using multivariable modeling and determined whether acute physiological parameters measured at ICU admission carry greater prognostic significance than baseline comorbidity burden. These findings may facilitate early risk stratification and inform clinical decision-making in geriatric medical intensive care units, particularly in resource-constrained healthcare environments.

## Materials and methods

Study design and setting

We conducted a single-center retrospective observational cohort study in the Geriatric Medical ICU of the Department of Geriatric Medicine at the All India Institute of Medical Sciences (AIIMS), New Delhi, India. This dedicated unit specializes exclusively in the management of critically ill older adults with complex medical illnesses. The study included all eligible patients admitted during a six-month period from June to December 2020. This window was selected because it represented the first complete six-month period for which fully digitized admission records for the dedicated geriatric medical ICU were available; the interval between data collection and publication reflects the time required for de-identification, record curation, and analysis. Owing to the retrospective design and use of fully anonymized data, no formal ethical approval was required by the Institutional Ethics Committee. Reporting follows the STROBE (Strengthening the Reporting of Observational Studies in Epidemiology) recommendations for cohort studies [13].

Participants and data collection

All consecutive patients aged ≥60 years admitted to the geriatric medical ICU during the study period were screened. Patients were included if their ICU stay exceeded two hours (to exclude brief observational or procedural admissions). Exclusion criteria were primary admission to another specialized ICU (surgical, cardiac, trauma, neurosurgical, or coronary care unit) with subsequent transfer; readmission during the same hospitalization (only the first admission analyzed); documented do-not-intubate (DNI) or comfort-care-only orders at admission; or COVID-19 patients. COVID-19 patients were excluded because, during the study period, they were managed in physically separate, dedicated COVID ICUs; their inclusion would have confounded the relationship between routine geriatric critical illness and the study outcomes. A total of 291 patients met the inclusion criteria and were included in the final analysis. Missing data were handled by complete-case analysis; variables with >10% missingness were not included in multivariable models. Data was extracted from the hospital’s electronic medical records using a predefined structured pro forma. Data extraction was performed by trained senior residents and independently verified by the principal investigator to ensure accuracy and completeness. Variables recorded at ICU admission included demographic characteristics, comorbidities, functional status, physiological parameters, and laboratory investigations.

Definitions of variables and outcomes

Multimorbidity was defined as the presence of two or more chronic medical conditions; baseline comorbidity burden was operationalized as multimorbidity together with individual documented comorbidities. Activities of daily living (ADL) dependence was defined as requiring assistance in one or more basic activities such as feeding, bathing, dressing, toileting, or transferring. Altered mental status was defined as impaired consciousness or disorientation at presentation. AKI was identified using the serum creatinine criterion of the 2012 KDIGO (Kidney Disease: Improving Global Outcomes) clinical practice guideline [14]. Septic shock was diagnosed using Sepsis-3 criteria: persistent circulatory dysfunction after volume resuscitation that required vasopressors to keep mean arterial pressure at 65 mmHg or above, together with a serum lactate above 2 mmol/L [15]. Hypoxemic respiratory failure at admission was defined as oxygen saturation <90% with arterial PaO₂ <60 mmHg on room air or supplemental oxygen. Acute liver injury (ALI) was defined as elevation of serum transaminases (AST or ALT >40 U/L) with clinical evidence of hepatic dysfunction. Dyselectrolytemia was defined as sodium <135 mmol/L or >145 mmol/L, or potassium <3.5 mmol/L or >5.0 mmol/L. 5 mmol/L or >5.0 mmol/L. 5 mmol/L or >5.0 mmol/L. Frailty was not systematically assessed using a validated tool (e.g., Clinical Frailty Scale) during the study period. The primary outcome was the requirement for invasive mechanical ventilation (IMV) at any point during an ICU stay, defined as endotracheal intubation with positive-pressure ventilation. Patients managed with non-invasive ventilation or high-flow nasal oxygen alone, without subsequent intubation, were classified as not having received IMV; those escalated from non-invasive support to endotracheal intubation were classified as having received IMV. The secondary outcome was in-hospital all-cause mortality, defined as death during the index hospitalization. Time-to-event was calculated from ICU admission until death or discharge.

Statistical analysis

No a priori sample-size calculation was performed; all consecutive eligible patients admitted during the study period were included (census sampling). The resulting 124 IMV events and 137 deaths provided approximately 25 events per candidate predictor in each final model, exceeding the conventional minimum of 10 events per variable. Continuous variables were assessed for normality using the Shapiro-Wilk test and visual inspection of histograms. All continuous variables were presented as medians with interquartile range (IQR) for consistency and clinical interpretability. Categorical variables were summarized as frequencies and percentages. Between group comparisons used the Mann-Whitney U test for continuous variables (after non-normality was confirmed) and the chi-square test with Yates' correction or Fisher's exact test for categorical variables, depending on expected cell counts.

Missing data were handled by available-case (complete-case) analysis. All candidate variables had less than 10% missingness, with the single exception of admission lactate, which was measured in 192 patients (66.0%) and missing in 99 (34.0%). Because of this substantial missingness, the primary multivariable mortality model excluded lactate and was fitted on the larger complete-covariate sample (n=265). Given its established prognostic relevance in critical illness, lactate was examined separately in a pre-specified secondary model restricted to patients with a measured admission lactate (n=186) and in univariable and Kaplan-Meier analyses; the reduced and potentially non-representative lactate subset is acknowledged as a limitation. The number of patients contributing to each model is reported with the corresponding results. To identify independent predictors of IMV, multivariable logistic regression was performed. All candidate predictors, those reaching p<0.10 on univariable testing together with variables judged clinically important a priori, were entered into a single full model, which was then reduced to a parsimonious model by backward elimination (removing the least significant variable at each step). The term “final model” denotes this single parsimonious model retained at the end of that process; no alternative or competing model specifications were fitted or compared, and the intermediate models were simply the successive backward-elimination steps. The univariable associations for every candidate predictor are reported alongside the retained multivariable model (variables not retained are shown as “-”). Results were expressed as adjusted odds ratios (ORs) with 95% confidence intervals (CIs); total leukocyte count was scaled per 1000 cells/µL. Predictors of in-hospital mortality were evaluated using a Cox proportional-hazards model, with hazard ratios (HR) and 95% CIs. The proportional-hazards assumption was assessed using Schoenfeld residuals and log-minus-log plots. Multicollinearity was assessed using variance inflation factors (VIF; values <3 acceptable).

Model discrimination for the ventilation model was evaluated using the area under the receiver operating characteristic curve (AUC-ROC), and calibration was assessed using the Hosmer-Lemeshow goodness-of-fit test. For the mortality model, predictive performance was assessed using Harrell’s concordance index (C-index). All analyses were performed using IBM Corp. Released 2020. IBM SPSS Statistics for Windows, Version 26. Armonk, NY: IBM Corp. To assess internal validity and model stability, bootstrap resampling with 200 iterations was performed for the logistic regression model, and optimism-corrected performance estimates were derived. Survival probabilities were estimated using the Kaplan-Meier method and compared using the log-rank test. Survival curves were stratified using clinically relevant thresholds for age (≥75 years) and admission lactate (≥2 mmol/L), based on clinical relevance and supported by receiver operating characteristic analysis. A two-sided p-value <0.05 was considered statistically significant.

## Results

During the study period, a total of 291 consecutive patients aged ≥60 years were admitted to the geriatric medical ICU and included in the analysis. The median age of the cohort was 76 years (IQR 69-80), and 176 patients (60.5%) were male. Invasive mechanical ventilation was required in 124 patients (42.6%), and in-hospital mortality was 47.1% (137 patients).

Baseline characteristics stratified by age

**Figure 1:**
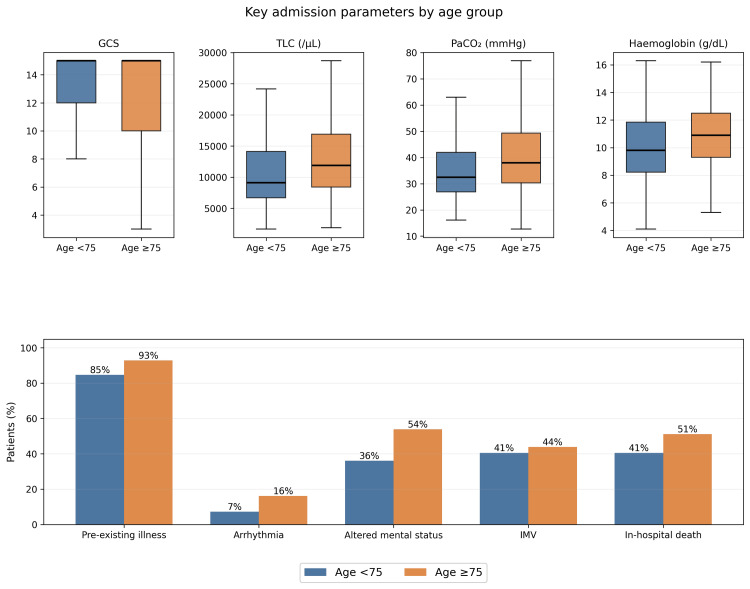
Graphical summary of the group comparisons by age Key admission parameters stratified by age (<75 versus ≥75 years). Upper panels: box plots (median, IQR, range) of selected continuous parameters. Lower panel: prevalence of comorbid illness, syndromes, and outcomes. Comprehensive comparisons in Table 1. GCS: Glasgow Coma Scale; IMV: invasive mechanical ventilation; IQR: interquartile range; PaCO₂: arterial partial pressure of carbon dioxide; TLC: total leukocyte count.

Patients were stratified into two clinically relevant age groups: <75 years (n=111, 38.1%) and ≥75 years (n=180, 61.9%) (Table 1, Figure 1). Patients aged ≥75 years had a significantly higher prevalence of pre-existing comorbidity excluding malignancy (92.8% vs. 84.7%, p=0.045) and a greater frequency of baseline arrhythmia (16.1% vs. 7.2%, p=0.042). Altered mental status was more frequent in the older group (53.9% vs. 36.0%, p=0.004), with a lower median Glasgow Coma Scale (GCS) score (15 [10-15] vs. 15 [12-15], p=0.020). Among laboratory parameters, patients ≥75 years demonstrated higher total leukocyte counts (median 11,900 vs. 9,100/µL, p=0.002), higher PaCO₂ levels (37.2 vs. 35.0 mmHg, p=0.023), and a greater prevalence of dyselectrolytemia (65.6% vs. 53.2%, p=0.048). Hemoglobin levels were higher in the older group (10.9 vs. 9.9 g/dL, p=0.007), while malignancy was less frequent (8.9% vs. 19.8%, p=0.012). There were no statistically significant differences between age groups in rates of invasive mechanical ventilation (43.9% vs. 40.5%, p=0.661) or in most major comorbid conditions, including diabetes, hypertension, coronary artery disease, and chronic obstructive airway disease. In-hospital mortality was numerically higher among patients aged ≥75 years, but the difference did not reach statistical significance (51.1% vs. 40.5%, p=0.102). Median ICU length of stay was shorter in the older group (9 vs. 11 days, p=0.004).

**Table 1:** Baseline characteristics of the study population stratified by age group (<75 vs. ≥75 years) Data are presented as medians (interquartile range) for continuous variables and numbers (percentages) for categorical variables. Continuous variables were compared using the Mann–Whitney U test (test statistic: U). Categorical variables were compared using the Pearson chi-square test or Fisher's exact test (test statistic: χ²). A two-sided p-value <0.05 was considered statistically significant; significant values are marked with an asterisk (*). ADL: activities of daily living; BP: blood pressure; GCS: Glasgow Coma Scale; RBS: random blood sugar; SpO₂: peripheral oxygen saturation; PaO₂: partial pressure of oxygen; PaCO₂: partial pressure of carbon dioxide; HCO₃: bicarbonate; Hb: hemoglobin; TLC: total leukocyte count; AST: aspartate aminotransferase; ALT: alanine aminotransferase; AKI: acute kidney injury.

Variable	Age <75 (n=111)	Age ≥75 (n=180)	Test statistic	p-value
Age (years), median (IQR)	67 (63–70)	79 (76–84)	U=0.0	<0.001*
Sex (male), n (%)	66 (59.5)	110 (61.1)	χ²=0.02	0.876
Diabetes mellitus, n (%)	44 (39.6)	67 (37.2)	χ²=0.08	0.773
Hypertension, n (%)	65 (59.1)	116 (64.4)	χ²=0.62	0.430
Coronary artery disease, n (%)	32 (28.8)	63 (35.0)	χ²=0.93	0.336
Chronic obstructive airway disease, n (%)	36 (32.4)	58 (32.2)	χ²=0.00	1.000
Heart failure, n (%)	19 (17.1)	35 (19.4)	χ²=0.12	0.733
Cerebrovascular accident, n (%)	15 (13.5)	35 (19.4)	χ²=1.31	0.253
Malignancy, n (%)	22 (19.8)	16 (8.9)	χ²=6.20	0.013*
Multimorbidity (≥2 comorbidities), n (%)	81 (73.0)	120 (66.7)	χ²=1.00	0.317
Pre-existing illness (any comorbidity excluding malignancy), n (%)	94 (84.7)	167 (92.8)	χ²=4.03	0.045*
ADL dependent, n (%)	30 (27.0)	66 (36.7)	χ²=2.47	0.116
Pulse rate (bpm), median (IQR)	91 (84–110)	98 (86–110)	U=9,260.0	0.295
Arrhythmia present, n (%)	8 (7.2)	29 (16.1)	χ²=4.14	0.042*
Systolic BP (mmHg), median (IQR)	114 (100–134)	120 (100–139)	U=9,521.0	0.552
Diastolic BP (mmHg), median (IQR)	70 (60–82)	69 (58–80)	U=10,959.0	0.140
Inotrope use, n (%)	21 (18.9)	44 (24.4)	χ²=0.91	0.340
Respiratory rate (breaths/min), median (IQR)	20 (18–26)	22 (18–26)	U=9,224.0	0.270
SpO₂ (%), median (IQR)	95 (90–97)	94 (89–97)	U=10,713.5	0.260
Febrile, n (%)	32 (28.8)	37 (20.6)	χ²=2.16	0.142
GCS, median (IQR)	15 (12–15)	15 (10–15)	U=11,349.0	0.017*
Altered mental status, n (%)	40 (36.0)	97 (53.9)	χ²=8.08	0.004*
RBS (mg/dL), median (IQR)	145 (112–184)	145 (116–188)	U=8,904.5	0.807
pH, median (IQR)	7.38 (7.30–7.44)	7.35 (7.25–7.41)	U=9,934.0	0.028*
PaO₂ (mmHg), median (IQR)	63.0 (46.4–92.3)	61.0 (40.2–87.2)	U=8,952.5	0.351
PaCO₂ (mmHg), median (IQR)	32.5 (26.9–42.0)	38.0 (30.3–49.3)	U=7,079.0	0.025*
HCO₃ (mEq/L), median (IQR)	20.4 (16.9–24.6)	20.0 (17.0–26.0)	U=8,260.5	0.865
Lactate (mmol/L), median (IQR)	1.6 (1.3–2.8)	1.5 (1.1–2.2)	U=4,618.5	0.150
Hb (g/dL), median (IQR)	9.8 (8.2–11.8)	10.9 (9.3–12.5)	U=7,992.5	0.007*
Platelets (×10⁵/µL), median (IQR)	1.9 (1.2–2.7)	1.8 (1.0–2.8)	U=10,783.5	0.148
TLC (/µL), median (IQR)	9,100 (6,685–14,120)	11,900 (8,400–16,900)	U=7,630.0	0.002*
Urea (mg/dL), median (IQR)	58 (33–98)	66 (39–124)	U=8,655.0	0.166
Creatinine (mg/dL), median (IQR)	1.3 (0.8–2.5)	1.5 (0.9–2.5)	U=9,188.0	0.604
Na⁺ (mEq/L), median (IQR)	136.8 (133.0–140.1)	137.0 (133.0–143.0)	U=8,980.0	0.327
K⁺ (mEq/L), median (IQR)	4.4 (3.9–4.8)	4.4 (3.7–4.8)	U=10,189.5	0.424
Albumin (g/dL), median (IQR)	2.9 (2.5–3.5)	2.9 (2.4–3.3)	U=9,254.5	0.558
AST (U/L), median (IQR)	33 (24–64)	32 (22–59)	U=8,920.0	0.716
ALT (U/L), median (IQR)	24 (14–44)	22 (14–43)	U=8,889.0	0.820
Hypoxaemic respiratory failure, n (%)	11 (9.9)	19 (10.6)	χ²=0.00	1.000
Acute liver injury, n (%)	46 (41.4)	70 (38.9)	χ²=0.10	0.758
Septic shock, n (%)	9 (8.1)	15 (8.3)	χ²=0.00	1.000
AKI, n (%)	56 (50.5)	101 (56.1)	χ²=0.67	0.412
Dyselectrolytaemia, n (%)	59 (53.2)	118 (65.6)	χ²=3.93	0.048*
Length of stay (days), median (IQR)	11 (7–20)	9 (5–15)	U=11,982.5	0.004*
Mechanical ventilation, n (%)	45 (40.5)	79 (43.9)	χ²=0.19	0.661
In-hospital death, n (%)	45 (40.5)	92 (51.1)	χ²=2.67	0.102

Baseline characteristics stratified by invasive mechanical ventilation

**Figure 2:**
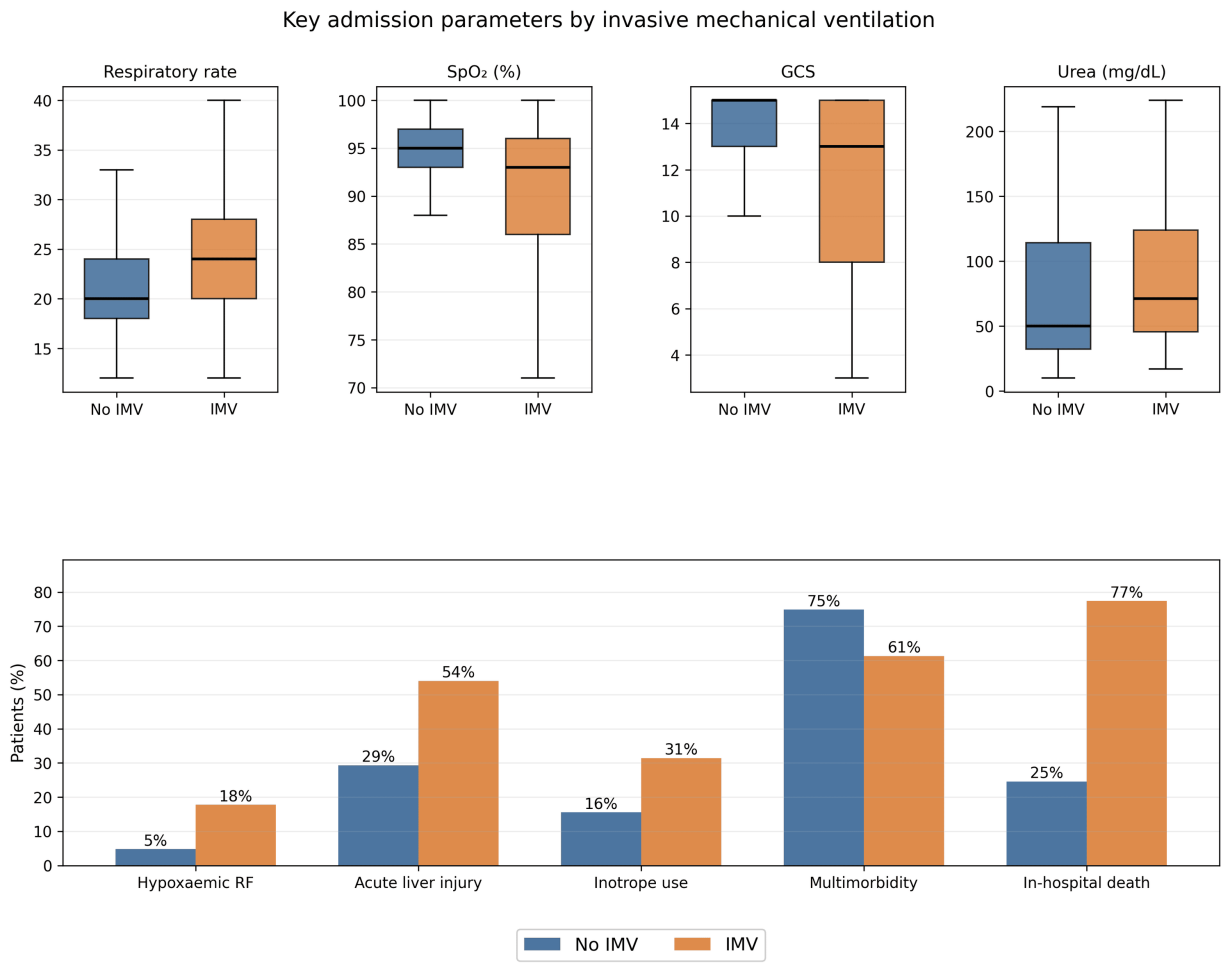
Graphical summary of the group comparisons by invasive mechanical ventilation Key admission parameters stratified by IMV requirement. Upper panels: acute physiological parameters. Lower panel: organ-dysfunction syndromes and outcomes. Acute physiology, not chronic burden, drove ventilation. Full comparisons in Table 2. GCS: Glasgow Coma Scale; IMV: invasive mechanical ventilation; IQR: interquartile range; RF: respiratory failure; SpO₂: peripheral oxygen saturation.

Invasive mechanical ventilation (IMV) was required in 124 patients (42.6%) (Table 2, Figure 2). At the time of ICU admission, ventilated patients had significantly higher respiratory rates (median 24 vs. 20 breaths/min, p<0.001), higher pulse rates (100 vs. 96 bpm, p=0.008), and lower oxygen saturation (93% vs. 95%, p<0.001). Neurological status differed significantly, with lower median GCS scores (13 vs. 15, p<0.001) and a higher prevalence of altered mental status (54.8% vs. 41.3%, p=0.030). Arterial blood gas analysis demonstrated lower pH (7.3 vs. 7.4, p<0.001) and higher lactate levels (p=0.032) among ventilated patients. Total leukocyte count was higher (median 12,530 vs. 9900/µL, p=0.007), as were urea levels (69.5 vs. 50 mg/dL, p=0.005). Liver enzymes were elevated (AST 38 vs. 30 U/L, p<0.001; ALT 26 vs. 20 U/L, p<0.001). Hypoxemic respiratory failure (17.7% vs. 4.8%, p<0.001), acute liver injury (54.0% vs. 29.3%, p<0.001), and inotrope use (31.5% vs. 15.6%, p=0.002) were more frequent among ventilated patients. Baseline demographic variables, including age and sex, and most comorbidities did not differ significantly between ventilated and non-ventilated groups. In-hospital mortality was markedly higher among patients who required invasive mechanical ventilation (77.4% vs. 24.6%, p<0.001). Malignancy was less frequent among ventilated patients (6.5% vs. 18.0%, p=0.007), and multimorbidity was lower (61.3% vs. 74.9%, p=0.019).

**Table 2:** Baseline characteristics stratified by invasive mechanical ventilation status Data are presented as medians (interquartile range) for continuous variables and numbers (percentages) for categorical variables. Continuous variables were compared using the Mann–Whitney U test (test statistic: U). Categorical variables were compared using the Pearson chi-square test or Fisher's exact test (test statistic: χ²). A two-sided p-value <0.05 was considered statistically significant; significant values are marked with an asterisk (*). ADL: activities of daily living; BP: blood pressure; GCS: Glasgow Coma Scale; SpO₂, peripheral oxygen saturation; PaO₂: partial pressure of oxygen; PaCO₂: partial pressure of carbon dioxide; HCO₃: bicarbonate; Hb: hemoglobin; TLC: total leukocyte count; AST: aspartate aminotransferase; ALT: alanine aminotransferase; AKI: acute kidney injury.

Variable	No IMV (n=167)	IMV (n=124)	Test statistic	p-value
Age (years), median (IQR)	76 (70–80)	76 (70–80)	U=10,083.0	0.703
Sex (male), n (%)	96 (57.5)	80 (64.5)	χ²=1.19	0.275
Diabetes mellitus, n (%)	69 (41.3)	42 (33.9)	χ²=1.37	0.242
Hypertension, n (%)	104 (62.7)	77 (62.1)	χ²=0.00	1.000
Coronary artery disease, n (%)	59 (35.3)	36 (29.0)	χ²=1.01	0.314
Chronic obstructive airway disease, n (%)	46 (27.5)	48 (38.7)	χ²=3.56	0.059
Heart failure, n (%)	35 (21.0)	19 (15.3)	χ²=1.15	0.284
Cerebrovascular accident, n (%)	28 (16.8)	22 (17.7)	χ²=0.00	0.951
Malignancy, n (%)	30 (18.0)	8 (6.5)	χ²=7.19	0.007*
Multimorbidity (≥2 comorbidities), n (%)	125 (74.9)	76 (61.3)	χ²=5.51	0.019*
Pre-existing illness (any comorbidity excluding malignancy), n (%)	150 (89.8)	111 (89.5)	χ²=0.00	1.000
ADL dependent, n (%)	56 (33.5)	40 (32.3)	χ²=0.01	0.918
Pulse rate (bpm), median (IQR)	96 (82–104)	100 (88–115)	U=8,473.5	0.008*
Arrhythmia present, n (%)	21 (12.6)	16 (12.9)	χ²=0.00	1.000
Systolic BP (mmHg), median (IQR)	120 (102–138)	114 (98–140)	U=10,980.0	0.315
Diastolic BP (mmHg), median (IQR)	70 (60–80)	66 (58–80)	U=11,308.5	0.141
Inotrope use, n (%)	26 (15.6)	39 (31.5)	χ²=9.45	0.002*
Respiratory rate (breaths/min), median (IQR)	20 (18–24)	24 (20–28)	U=7,804.0	<0.001*
SpO₂ (%), median (IQR)	95 (93–97)	93 (86–96)	U=12,689.0	<0.001*
Febrile, n (%)	37 (22.2)	32 (25.8)	χ²=0.34	0.559
GCS, median (IQR)	15 (13–15)	13 (8–15)	U=13,123.0	<0.001*
Altered mental status, n (%)	69 (41.3)	68 (54.8)	χ²=4.69	0.030*
RBS (mg/dL), median (IQR)	142 (114–184)	146 (109–186)	U=9,426.0	0.822
pH, median (IQR)	7.38 (7.32–7.43)	7.33 (7.23–7.40)	U=11,386.0	<0.001*
PaO₂ (mmHg), median (IQR)	61.5 (42.2–87.2)	62.5 (41.4–91.9)	U=8,763.5	0.735
PaCO₂ (mmHg), median (IQR)	34.8 (28.2–42.8)	38.3 (28.3–56.2)	U=7,871.0	0.065
HCO₃ (mEq/L), median (IQR)	20.3 (17.4–25.3)	20.4 (16.3–25.6)	U=9,146.0	0.718
Lactate (mmol/L), median (IQR)	1.5 (1.1–2.2)	1.7 (1.2–2.7)	U=3,888.5	0.062
Hb (g/dL), median (IQR)	10.6 (8.9–12.1)	10.7 (8.4–12.5)	U=10,148.5	0.932
Platelets (×10⁵/µL), median (IQR)	1.9 (1.2–2.8)	1.7 (1.0–2.6)	U=10,895.0	0.271
TLC (/µL), median (IQR)	9,900 (6,860–14,660)	12,530 (8,425–19,292)	U=8,205.5	0.007*
Urea (mg/dL), median (IQR)	50 (32–114)	71 (46–124)	U=7,975.5	0.005*
Creatinine (mg/dL), median (IQR)	1.3 (0.8–2.4)	1.5 (1.0–2.7)	U=8,658.0	0.084
Na⁺ (mEq/L), median (IQR)	136.7 (133.0–141.0)	138.0 (133.1–144.0)	U=8,919.0	0.124
K⁺ (mEq/L), median (IQR)	4.3 (3.9–4.8)	4.4 (3.7–4.9)	U=9,965.0	0.980
Albumin (g/dL), median (IQR)	2.9 (2.5–3.4)	2.9 (2.4–3.2)	U=9,908.5	0.176
AST (U/L), median (IQR)	28 (20–48)	44 (25–66)	U=6,262.0	<0.001*
ALT (U/L), median (IQR)	18 (13–37)	30 (18–52)	U=6,784.0	<0.001*
Hypoxaemic respiratory failure, n (%)	8 (4.8)	22 (17.7)	χ²=11.55	<0.001*
Acute liver injury, n (%)	49 (29.3)	67 (54.0)	χ²=17.08	<0.001*
Septic shock, n (%)	9 (5.4)	15 (12.1)	χ²=3.39	0.066
AKI, n (%)	84 (50.3)	73 (58.9)	χ²=1.77	0.183
Dyselectrolytaemia, n (%)	94 (56.3)	83 (66.9)	χ²=2.95	0.086
Length of stay (days), median (IQR)	10 (6–14)	11 (5–19)	U=9,867.0	0.493
In-hospital death, n (%)	41 (24.6)	96 (77.4)	χ²=77.73	<0.001*

Baseline characteristics stratified by survival

**Figure 3:**
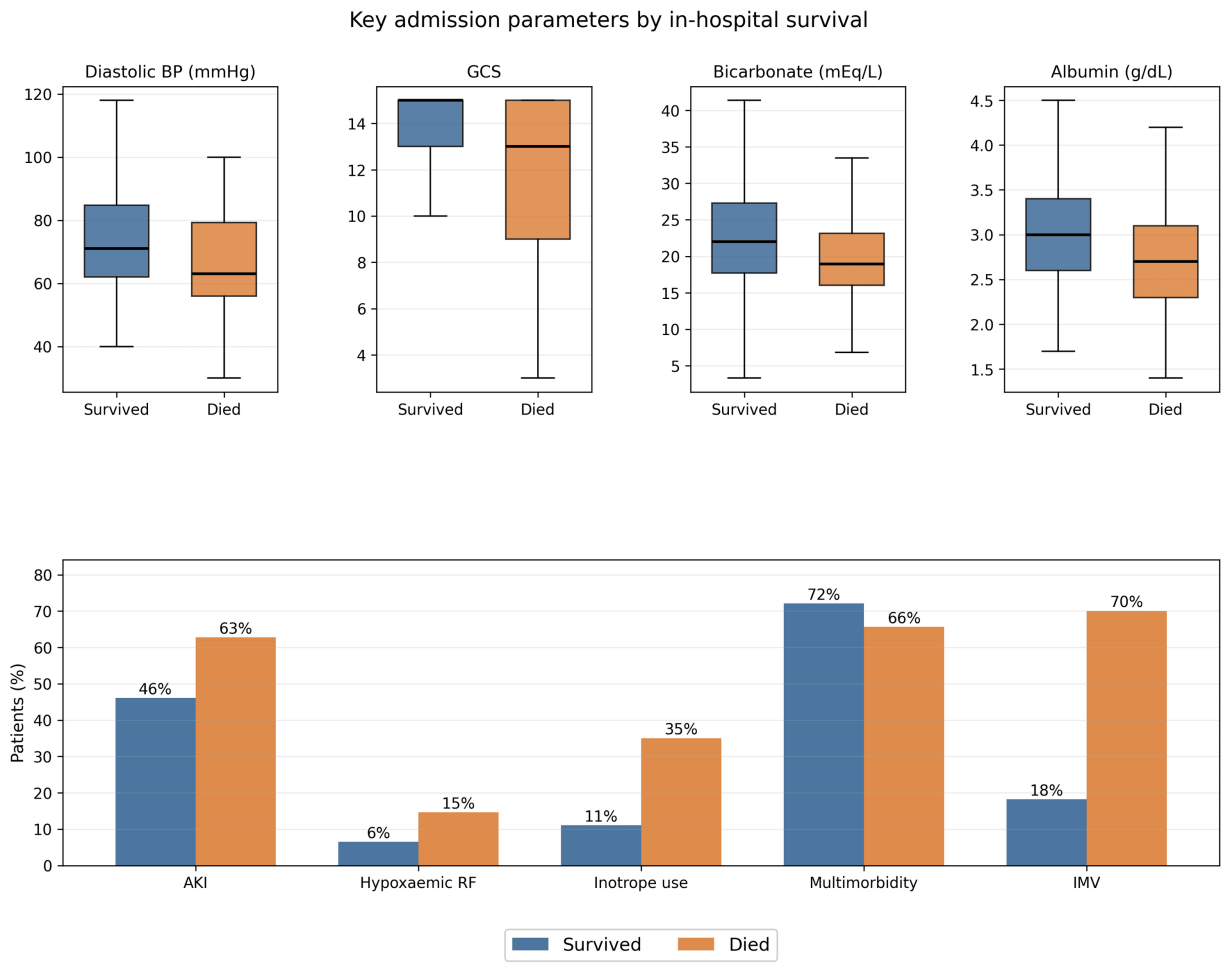
Graphical summary of the group comparisons by in-hospital survival Key admission parameters stratified by survival. Upper panels: hemodynamic and metabolic parameters. Lower panel: syndromes and interventions. Chronic disease burden did not distinguish the groups. Comprehensive comparisons in Table 3. AKI: acute kidney injury; GCS: Glasgow Coma Scale; IMV: invasive mechanical ventilation; IQR: interquartile range; RF: respiratory failure.

Overall in-hospital mortality was 47.1% (137/291) (Table 3, Figure 3). Non-survivors had significantly lower systolic blood pressure (110 vs. 122 mmHg, p<0.001) and diastolic blood pressure (63 vs. 71 mmHg, p<0.001) at admission. Inotrope use was more frequent among non-survivors (35.0% vs. 11.0%, p<0.001). Neurological status was significantly impaired among non-survivors, with lower median GCS scores (13 vs. 15, p<0.001) and a higher prevalence of altered mental status (56.9% vs. 38.3%, p=0.002). Laboratory parameters demonstrated higher lactate levels (p<0.001), lower bicarbonate levels (19.0 vs. 20.6 mEq/L, p<0.001), higher urea (74 vs. 49 mg/dL, p<0.001), higher creatinine (1.6 vs. 1.2 mg/dL, p<0.001), and lower albumin levels (2.9 vs. 3.0 g/dL, p<0.001) in non-survivors. Total leukocyte count was also higher (11,900 vs. 9,500/µL, p=0.013). Acute kidney injury (62.8% vs. 46.1%, p=0.006), acute liver injury (48.9% vs. 31.8%, p=0.004), septic shock (13.1% vs. 3.9%, p=0.008), and hypoxemic respiratory failure (14.6% vs. 6.5%, p=0.038) were more frequent among non-survivors. Invasive mechanical ventilation was required in 70.1% of non-survivors compared with 18.2% of survivors (p<0.001). Median hospital length of stay was shorter among non-survivors (8 vs. 11 days, p<0.001). There were no statistically significant differences in age, sex, or most baseline comorbidities between survivors and non-survivors.

**Table 3:** Baseline characteristics stratified by in-hospital outcome (survivors vs. non-survivors) Data are presented as medians (interquartile range) for continuous variables and numbers (percentages) for categorical variables. Continuous variables were compared using the Mann–Whitney U test (test statistic: U). Categorical variables were compared using the Pearson chi-square test or Fisher's exact test (test statistic: χ²). A two-sided P value <0.05 was considered statistically significant; significant values are marked with an asterisk (*). ADL: activities of daily living; BP: blood pressure; GCS: Glasgow Coma Scale; SpO₂: peripheral oxygen saturation; PaO₂: partial pressure of oxygen; PaCO₂: partial pressure of carbon dioxide; HCO₃: bicarbonate; Hb: hemoglobin; TLC: total leukocyte count; AST: aspartate aminotransferase; ALT: alanine aminotransferase; AKI: acute kidney injury.

Variable	Survivors (n=154)	Non-survivors (n=137)	Test statistic	p-value
Age (years), median (IQR)	75 (68–80)	77 (70–80)	U=9,515.5	0.149
Sex (male), n (%)	93 (60.4)	83 (60.6)	χ²=0.00	1.000
Diabetes mellitus, n (%)	56 (36.4)	55 (40.1)	χ²=0.29	0.588
Hypertension, n (%)	99 (64.7)	82 (59.9)	χ²=0.53	0.465
Coronary artery disease, n (%)	55 (35.7)	40 (29.2)	χ²=1.12	0.290
Chronic obstructive airway disease, n (%)	53 (34.4)	41 (29.9)	χ²=0.48	0.489
Heart failure, n (%)	33 (21.4)	21 (15.3)	χ²=1.40	0.236
Cerebrovascular accident, n (%)	24 (15.6)	26 (19.0)	χ²=0.37	0.542
Malignancy, n (%)	17 (11.0)	21 (15.4)	χ²=0.87	0.350
Multimorbidity (≥2 comorbidities), n (%)	111 (72.1)	90 (65.7)	χ²=1.10	0.294
Pre-existing illness (any comorbidity excluding malignancy), n (%)	136 (88.3)	125 (91.2)	χ²=0.39	0.531
ADL dependent, n (%)	46 (29.9)	50 (36.5)	χ²=1.16	0.282
Pulse rate (bpm), median (IQR)	96 (83–106)	100 (88–114)	U=9,341.5	0.092
Arrhythmia present, n (%)	21 (13.6)	16 (11.7)	χ²=0.11	0.746
Systolic BP (mmHg), median (IQR)	122 (107–140)	110 (90–130)	U=13,016.5	<0.001*
Diastolic BP (mmHg), median (IQR)	71 (62–85)	63 (56–79)	U=13,335.0	<0.001*
Inotrope use, n (%)	17 (11.0)	48 (35.0)	χ²=22.70	<0.001*
Respiratory rate (breaths/min), median (IQR)	20 (18–24)	22 (18–28)	U=9,180.5	0.055
SpO₂ (%), median (IQR)	95 (92–97)	94 (88–98)	U=11,319.0	0.233
Febrile, n (%)	43 (27.9)	26 (19.0)	χ²=2.73	0.098
GCS, median (IQR)	15 (13–15)	13 (9–15)	U=13,425.0	<0.001*
Altered mental status, n (%)	59 (38.3)	78 (56.9)	χ²=9.36	0.002*
RBS (mg/dL), median (IQR)	144 (114–187)	145 (115–186)	U=9,912.5	0.780
pH, median (IQR)	7.38 (7.32–7.43)	7.34 (7.23–7.40)	U=11,167.0	0.003*
PaO₂ (mmHg), median (IQR)	62.7 (43.3–91.3)	61.5 (41.0–88.2)	U=9,172.5	0.841
PaCO₂ (mmHg), median (IQR)	35.5 (28.9–48.4)	36.5 (27.3–45.7)	U=9,519.0	0.526
HCO₃ (mEq/L), median (IQR)	22.0 (17.7–27.3)	18.9 (16.0–23.1)	U=11,288.5	<0.001*
Lactate (mmol/L), median (IQR)	1.4 (1.1–1.9)	1.8 (1.2–2.7)	U=3,257.5	<0.001*
Hb (g/dL), median (IQR)	10.6 (9.0–12.4)	10.7 (8.4–12.2)	U=10,804.0	0.573
Platelets (×10⁵/µL), median (IQR)	1.9 (1.2–2.9)	1.6 (1.0–2.5)	U=11,882.5	0.028*
TLC (/µL), median (IQR)	9,500 (6,800–14,890)	11,900 (8,400–17,630)	U=8,512.0	0.013*
Urea (mg/dL), median (IQR)	49 (31–83)	76 (48–128)	U=6,861.5	<0.001*
Creatinine (mg/dL), median (IQR)	1.2 (0.8–2.0)	1.6 (1.0–3.2)	U=7,585.0	<0.001*
Na⁺ (mEq/L), median (IQR)	137.0 (133.0–141.0)	137.0 (132.0–143.0)	U=10,109.0	0.926
K⁺ (mEq/L), median (IQR)	4.4 (3.9–4.8)	4.4 (3.6–4.9)	U=10,555.5	0.585
Albumin (g/dL), median (IQR)	3.0 (2.6–3.4)	2.7 (2.3–3.1)	U=11,384.5	<0.001*
AST (U/L), median (IQR)	28 (21–46)	44 (24–75)	U=6,610.5	<0.001*
ALT (U/L), median (IQR)	19 (14–38)	28 (16–47)	U=7,638.5	0.027*
Hypoxaemic respiratory failure, n (%)	10 (6.5)	20 (14.6)	χ²=4.31	0.038*
Acute liver injury, n (%)	49 (31.8)	67 (48.9)	χ²=8.13	0.004*
Septic shock, n (%)	6 (3.9)	18 (13.1)	χ²=7.01	0.008*
AKI, n (%)	71 (46.1)	86 (62.8)	χ²=7.45	0.006*
Dyselectrolytaemia, n (%)	86 (55.8)	91 (66.4)	χ²=2.98	0.085
Length of stay (days), median (IQR)	11 (7–17)	8 (3–16)	U=13,259.0	<0.001*
Mechanical ventilation, n (%)	28 (18.2)	96 (70.1)	χ²=77.73	<0.001*

Multivariable predictors of invasive mechanical ventilation

**Figure 4:**
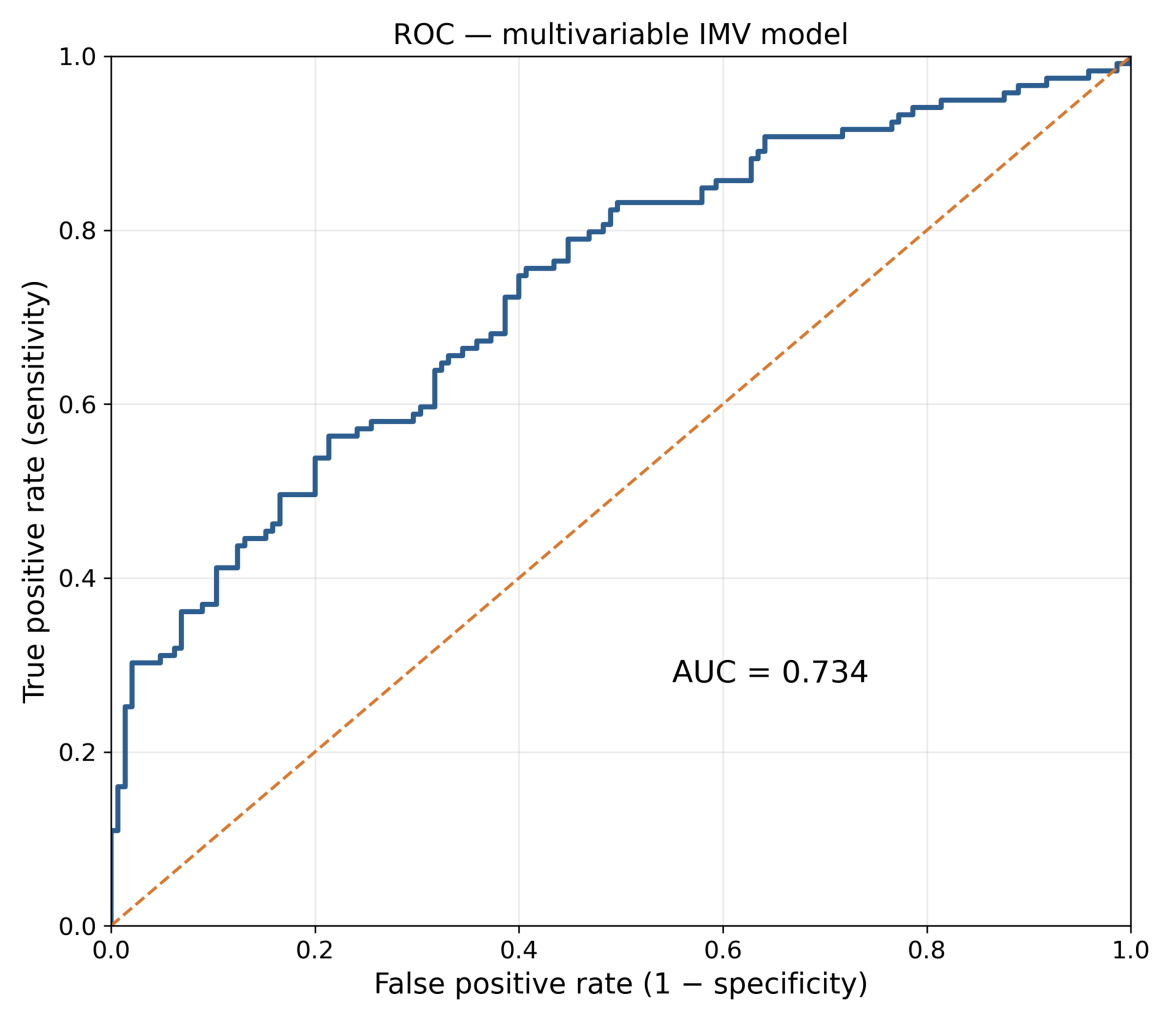
Receiver operating characteristic (ROC) curve of the logistic regression model for prediction of invasive mechanical ventilation ROC curve for the full multivariable logistic regression model combining all five candidate predictors (respiratory rate, SpO₂, GCS, PaCO₂, and total leukocyte count), fitted on n=264 patients with 119 events; the AUC for this composite model is 0.73 (95% CI 0.67–0.79).

Univariable and multivariable logistic regression results are presented in Table 4. On multivariable analysis, four variables were independently associated with IMV: respiratory rate (aOR 1.05, 95% CI 1.01-1.10, p=0.024), oxygen saturation (aOR 0.94, 0.91-0.98, p=0.008), Glasgow Coma Scale (aOR 0.84, 0.78-0.92, p<0.001), and PaCO₂ (aOR 1.02, 1.00-1.04, p=0.016). There was no evidence of multicollinearity (all VIFs <1.1). The model showed acceptable discrimination (AUC 0.73, 95% CI 0.67-0.79; Figure 4) and good calibration (Hosmer-Lemeshow χ²=3.71, df=8, p=0.882); McFadden pseudo-R²=0.14, log-likelihood −156.04, and AIC 324.1. The model was fitted on 264 patients (90.7%) with complete data for all included covariates. The model was fitted on 264 patients (90.7%) with complete data for all included covariates.

**Table 4:** Univariable and multivariable logistic regression analysis for predictors of invasive mechanical ventilation Odds ratios (OR) with 95% confidence intervals (CI) are presented. Variables significant in univariable analysis were considered for multivariable modeling. The test statistic is the Wald χ² (df=1) for each coefficient. A two-sided p value <0.05 was considered statistically significant; significant values are marked with an asterisk (*). Total leukocyte count (TLC) is modeled per 1000 cells/μL increase. Multicollinearity was assessed using the variance inflation factor (VIF, all <1.1). Model (n=264): McFadden pseudo-R²=0.14; log-likelihood=−156.04; AIC=324.1; AUC=0.73 (95% CI 0.67–0.79); Hosmer–Lemeshow χ²=3.71, df=8, p=0.882.

Variable	OR	95% CI	Wald χ²	P value	aOR	95% CI	Wald χ²	p-value
Chronic obstructive airway disease	1.66	1.01–2.73	4.03	0.045	—	—	—	—
Pulse rate	1.01	1.00–1.03	6.39	0.011	—	—	—	—
Inotrope use	2.49	1.41–4.38	10.02	0.002	—	—	—	—
Respiratory rate	1.08	1.04–1.13	14.32	<0.001	1.05	1.01–1.10	5.06	0.024
SpO₂	0.92	0.88–0.96	18.12	<0.001	0.94	0.91–0.98	7.12	0.008
GCS	0.82	0.76–0.89	24.48	<0.001	0.84	0.78–0.92	15.68	<0.001
Altered mental status	1.72	1.08–2.76	5.19	0.023	—	—	—	—
pH	0.01	0.00–0.13	13.40	<0.001	—	—	—	—
PaCO₂	1.02	1.01–1.04	8.51	0.004	1.02	1.00–1.04	5.78	0.016
Lactate	1.24	1.03–1.51	5.03	0.025	—	—	—	—
TLC (per 1000 cells/µL)	1.03	1.00–1.06	4.90	0.027	1.03	1.00–1.06	2.86	0.091

Multivariable predictors of in-hospital mortality

**Figure 5:**
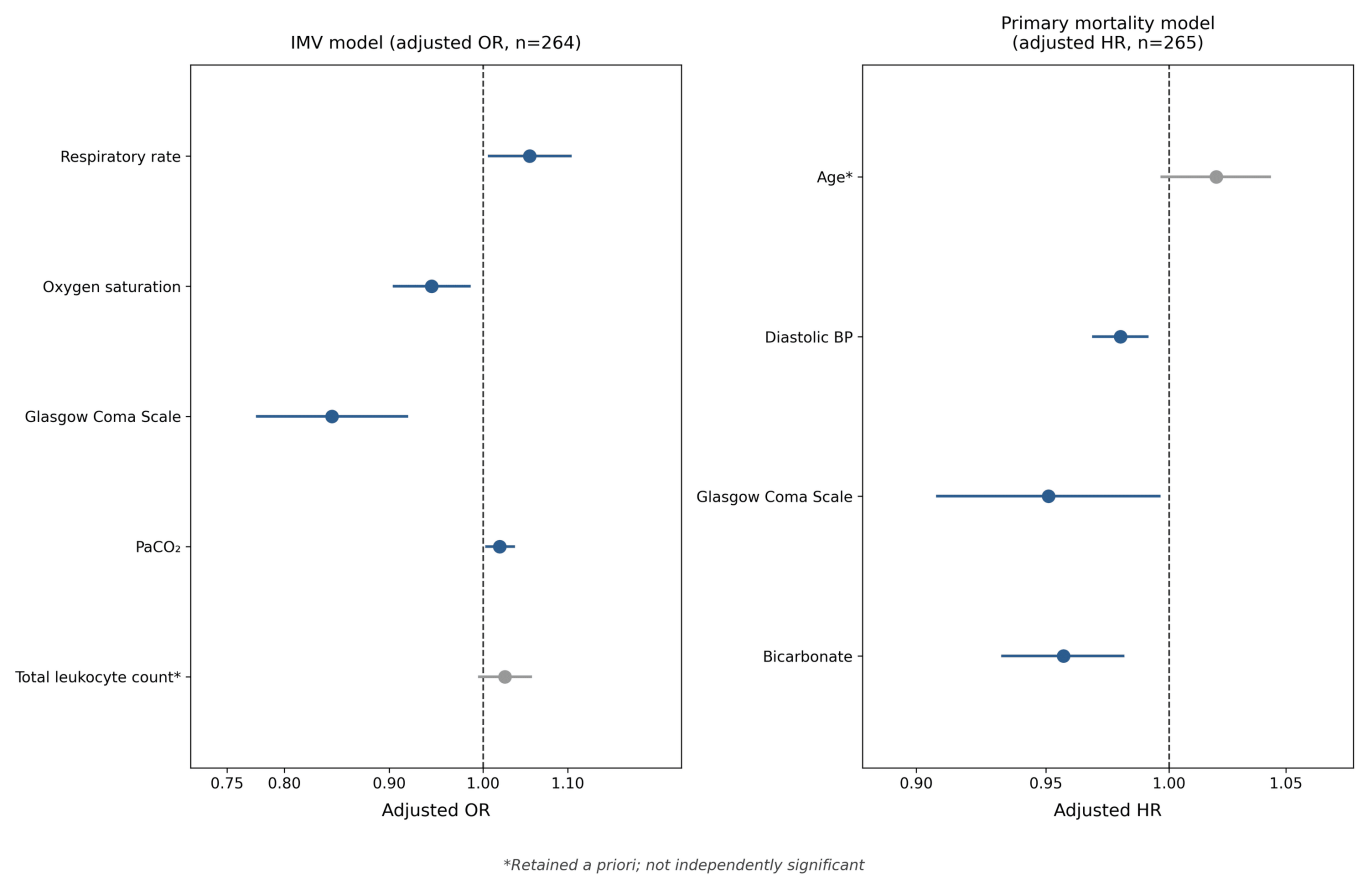
Forest plot of multivariable adjusted estimates for the IMV model and the primary mortality model Forest plot of multivariable adjusted estimates for the IMV model (left, aORs) and the primary mortality model (right, aHRs). Blue = independent predictors; grey = retained a priori, not independently significant. Left (n=264): RR (aOR 1.05, 1.01–1.10), SpO₂ (0.94, 0.91–0.98), GCS (0.84, 0.78–0.92), PaCO₂ (1.02, 1.00–1.04); TLC not significant (1.03). AUC 0.73 (0.67–0.79). Right (n=265): DBP (aHR 0.98, 0.97–0.99), GCS (0.95, 0.91–1.00), bicarbonate (0.96, 0.93–0.98); age not significant (1.02). C-index 0.70. Secondary lactate model (n=186): aHR 1.22 (1.10–1.36), C-index 0.73 — Table 6. Full results in Tables 4–5. aHR, adjusted hazard ratio; aOR, adjusted odds ratio; AUC, area under the ROC curve; CI, confidence interval; GCS, Glasgow Coma Scale; IMV, invasive mechanical ventilation; PaCO₂, arterial partial pressure of carbon dioxide; SpO₂, peripheral oxygen saturation; TLC, total leukocyte count (per 1000 cells/µL).

Univariable and multivariable Cox proportional-hazards results are shown in Table 5. Because admission lactate had 34.0% missingness, the primary multivariable model excluded it and was fitted on 265 patients (131 deaths). Three acute physiological variables were independently associated with mortality: diastolic blood pressure (aHR 0.98, 95% CI 0.97-0.99, p<0.001), bicarbonate (aHR 0.96, 0.93-0.98, p<0.001), and Glasgow Coma Scale (aHR 0.95, 0.91-1.00, p=0.035). Age, retained for clinical relevance and significant on univariable analysis, was not independently associated with mortality after adjustment (aHR 1.02, 1.00-1.04, p=0.082). VIFs were ≤1.1; the proportional-hazards assumption was not violated. The model demonstrated moderate discrimination (C-index 0.70). Because discharge alive was treated as censoring, this analysis does not formally account for discharge as a competing event (refer to limitations). Figure 5 shows the forest plot of multivariable adjusted estimates for the IMV model and the primary mortality model

**Table 5:** Univariable and multivariable Cox proportional-hazards analysis for predictors of in-hospital mortality (primary model, excluding lactate) Hazard ratios (HR) with 95% confidence intervals (CI) are reported. Variables significant in univariable analysis were included in multivariable Cox regression. The test statistic is the Wald χ² (df=1) for each coefficient. A two-sided p-value <0.05 was considered statistically significant; significant values are marked with an asterisk (*). The proportional hazards assumption was assessed using Schoenfeld residuals and was not violated. Multicollinearity was assessed using VIF (variance inflation factor; all ≤1.08). This is the primary mortality model and excludes admission lactate (34% missing); the lactate-containing secondary model is shown in Table 6. Age, significant on univariable analysis, was not independently associated with mortality after adjustment (aHR 1.02, p=0.082) and is retained for transparency. Model (n=265, 131 deaths): C-index=0.70.

Variable	HR	95% CI	Wald χ²	p-value	aHR	95% CI	Wald χ²	p-value
Age	1.03	1.00–1.05	5.34	0.021	1.02	1.00–1.04	3.03	0.082
Systolic BP	0.99	0.98–1.00	10.38	0.001	—	—	—	—
Diastolic BP	0.98	0.96–0.99	18.99	<0.001	0.98	0.97–0.99	11.97	<0.001
Inotrope use	1.89	1.32–2.69	12.16	<0.001	—	—	—	—
SpO₂	0.97	0.95–0.99	7.57	0.006	—	—	—	—
GCS	0.92	0.88–0.96	14.31	<0.001	0.95	0.91–1.00	4.45	0.035
Altered mental status	1.59	1.13–2.23	7.12	0.008	—	—	—	—
pH	0.07	0.02–0.30	12.67	<0.001	—	—	—	—
HCO₃	0.96	0.93–0.98	11.04	<0.001	0.96	0.93–0.98	12.06	<0.001
Lactate	1.27	1.15–1.40	23.59	<0.001	see Table 6	—	—	—
Platelets	0.82	0.71–0.95	7.08	0.008	—	—	—	—
Urea	1.00	1.00–1.00	9.13	0.003	—	—	—	—
Albumin	0.71	0.52–0.96	4.95	0.026	—	—	—	—
AST	1.00	1.00–1.00	9.58	0.002	—	—	—	—

Secondary analysis: admission lactate

**Figure 6:**
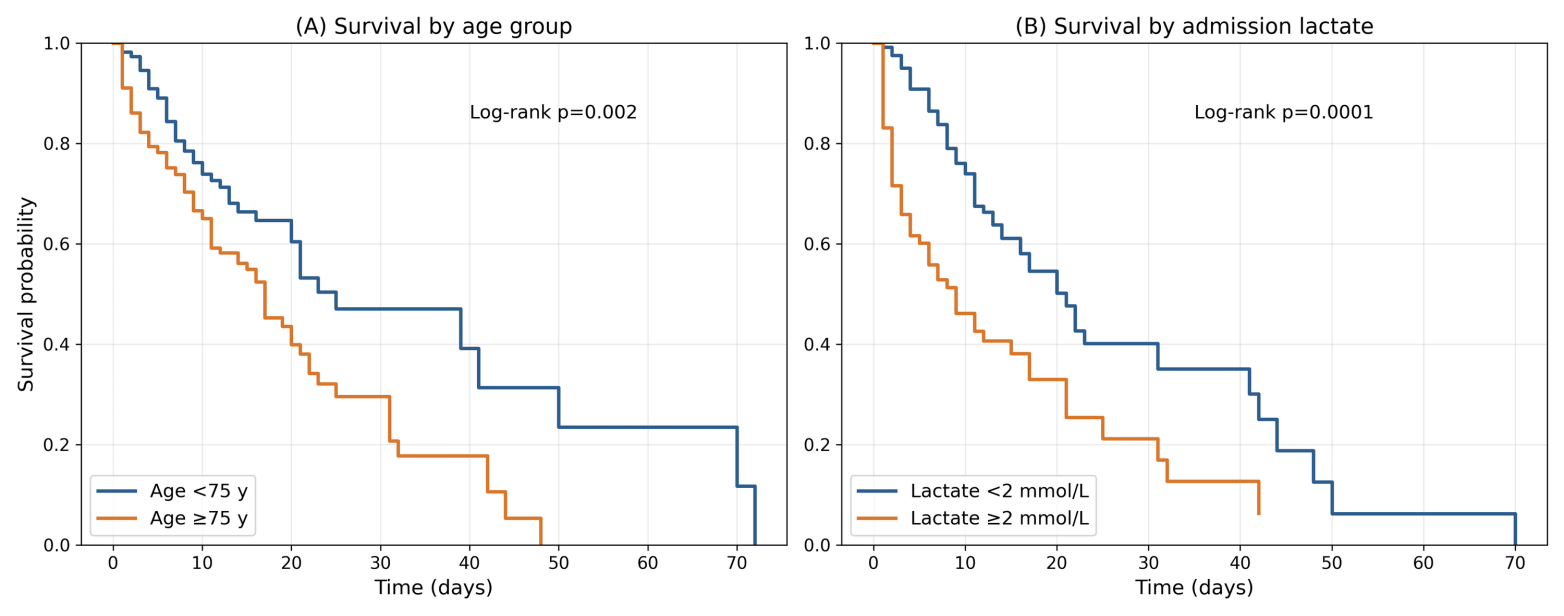
Kaplan–Meier survival curves stratified by (A) age and (B) admission lactate level Kaplan–Meier survival curves stratified by (A) age (<75 vs. ≥75 years; log-rank p=0.002) and (B) admission lactate (<2 vs. ≥2 mmol/L; log-rank p<0.0001).

Admission lactate was strongly associated with mortality wherever it was available. On univariable analysis, each 1 mmol/L increment was associated with a 27% higher hazard of death (HR 1.27, 95% CI 1.15-1.40, p<0.001), and Kaplan-Meier analysis showed markedly reduced survival above 2 mmol/L (Figure 6B; log-rank p<0.0001). In a secondary multivariable model restricted to the 186 patients (102 deaths) with a measured admission lactate, lactate remained independently associated with mortality (aHR 1.22, 95% CI 1.10-1.36, p<0.001) after adjustment for age, diastolic blood pressure, GCS, and bicarbonate, all of which retained their direction (Table 6; C-index 0.73). Because lactate is typically measured in patients judged more acutely unwell, this subset may be enriched for severity, and the secondary estimate should be interpreted as complementary to, rather than a replacement for, the primary model. Reassuringly, the independent associations of diastolic blood pressure, GCS, and bicarbonate were essentially unchanged between the primary (n=265) and lactate-containing (n=186) models, indicating that the central finding does not depend on the lactate subset.

**Table 6:** Secondary Cox proportional-hazards model including admission lactate Adjusted hazard ratios (aHR) with 95% CIs from the secondary Cox model that adds admission lactate, fitted on the 186 patients (102 deaths) with measured lactate. The test statistic is the Wald χ² (df=1) for each coefficient. A two-sided p-value <0.05 was considered statistically significant; significant values are marked with an asterisk (*). The proportional hazards assumption was assessed using Schoenfeld residuals and was not violated. Multicollinearity was assessed using VIF (variance inflation factor; all ≤1.08). Estimates are reported to two decimal places. C-index = 0.73.

Variable	aHR	95% CI	Wald χ²	p-value
Age	1.02	0.99–1.04	1.61	0.21
Diastolic BP	0.99	0.97–1.00	5.55	0.018
GCS	0.93	0.88–0.98	7.21	0.007
HCO₃	0.95	0.92–0.98	13.20	<0.001
Lactate	1.22	1.10–1.36	13.48	<0.001

Survival analysis

Kaplan-Meier survival curves are shown in Figure 6. Survival differed between age groups, with patients aged ≥75 years showing lower survival than those <75 years (log-rank p=0.002). Patients with admission lactate ≥2 mmol/L had markedly reduced survival compared with those with lower lactate (log-rank p<0.0001); the lactate-stratified analysis was restricted to the 192 patients (66.0%) with a measured admission lactate (71 with lactate ≥2 mmol/L).

## Discussion

In this retrospective cohort study of 291 older adults admitted to a dedicated geriatric medical ICU, acute physiological derangements at presentation were the principal determinant of both invasive mechanical ventilation and in-hospital mortality, whereas baseline comorbidity burden had limited independent prognostic significance. This pattern was consistent across stratified comparisons, multivariable modeling, and survival analysis, and aligns with global and LMIC cohorts in which outcomes in critically ill older adults are driven predominantly by acute illness severity rather than chronic disease burden [8,11]. The observed rates of IMV (42.6%) and in-hospital mortality (47.1%) align with contemporary Indian and international geriatric ICU data. Although crude in-hospital mortality was numerically higher among patients aged ≥75 years, this difference was not statistically significant (51.1% vs. 40.5%, p=0.102), and IMV rates were similar across age groups. After multivariable adjustment, age was not independently associated with mortality (aHR 1.02, p=0.21), indicating that any apparent age-mortality association was explained by the greater acute physiological derangement in older patients rather than by chronological age itself. Recent Indian studies report ICU mortality of 20-36% among older adults, rising to 40-50% in medically complex sepsis/multi-organ-dysfunction cases; one LMIC cohort reported 42.9% overall mortality, and the VIP-1/VIP-2 studies show 30-day mortality of 25-38% in patients ≥80 years [7,8,10,16]. These data suggest outcomes in a dedicated geriatric ICU in India are comparable to international benchmarks once patients access tertiary care.

In our cohort, patients aged ≥75 years had greater comorbidity burden, more arrhythmias, more altered mental status, and higher inflammatory markers, yet age was not associated with increased IMV requirement. The modest adjusted effect of age is consistent with the VIP studies, in which frailty and acute illness severity, rather than chronological age, dominated outcome prediction [11], and with Bagshaw et al., who showed that organ-failure severity outweighs chronological age [17]. Once critical illness develops, the magnitude of physiological derangement appears to outweigh baseline age-related changes, such that age carried no independent prognostic weight in our adjusted model.

Patients requiring IMV presented with marked derangement at admission, tachypnea, hypoxemia, hypercapnia, reduced GCS, and leukocytosis, consistent with established physiological indications for intubation [18]. In older adults, these mechanisms are amplified by age-related declines in respiratory muscle strength, chest-wall compliance, and ventilatory response to hypercapnia [19]. IMV itself was strongly associated with mortality (77.4% vs. 24.6%, p<0.001), serving as both a marker of severe illness and an independent predictor of poor outcome. Leukocytosis was associated with the need for IMV on univariable analysis, likely reflecting infection-driven respiratory failure from pneumonia or sepsis, the predominant causes of ICU admission in LMIC settings, with inflammaging a plausible contributor [20], although it did not reach independent significance after adjustment for the other physiological parameters (aOR 1.03, p=0.09). Chronic comorbidities were not independently associated with IMV, consistent with literature showing comorbidity burden has limited short-term predictive value compared with acute derangement [21,22]. The lower prevalence of malignancy among ventilated patients likely reflects treatment-limitation and triage decisions [23], underscoring clinical decision-making as a modifier of observed associations.

Non-survivors exhibited a phenotype of circulatory failure and tissue hypoperfusion, hemodynamic instability, low diastolic blood pressure, neurological impairment, metabolic acidosis with elevated lactate, and multi-organ dysfunction. These align with circulatory failure and progressive organ dysfunction as predominant drivers of mortality [24]. In older adults, arterial stiffening increases dependence on diastolic pressure for coronary perfusion, so diastolic hypotension directly reflects impaired organ perfusion; the resulting hypoperfusion drives lactate accumulation and metabolic acidosis [25]. The independent association of admission lactate with mortality (aHR 1.22, in the subset where it was measured) aligns with geriatric ICU literature in which modest lactate elevations independently predict short-term death [26]. Multivariable modeling identified four readily available admission parameters independently predicting IMV (respiratory rate, oxygen saturation, GCS, and PaCO₂; AUC 0.73) and three independently predicting mortality in the primary model (diastolic BP, bicarbonate, and GCS; n=265, C-index 0.70), with admission lactate adding further independent prognostic value in the subset in which it was available. Chronological age and total leukocyte count, although associated on univariable testing, were not independent predictors after adjustment. These encapsulate the core pathophysiology of critical illness in older adults [27]. All predictors can be obtained within minutes at the bedside, supporting immediate risk stratification and timely goals-of-care decisions where formal frailty assessment is impractical.

This study has several strengths: it is one of the few conducted exclusively in a dedicated geriatric medical ICU in India, with a relatively large, homogeneous cohort and a rigorous statistical approach (multivariable logistic and Cox modeling, backward elimination, bootstrap validation, multicollinearity and proportional-hazards checks, discrimination, and calibration). Several limitations must be acknowledged. The retrospective, single-center design from a tertiary referral hospital carries risks of selection bias and may not generalize to mixed medical-surgical ICUs, community or secondary-level hospitals, or non-specialized units. Frailty was not assessed with a validated instrument; because unmeasured frailty may both increase acute physiological derangement and influence the intensity of organ support offered, it may confound the observed associations and could account for part of the prognostic signal we attribute to acute physiology. Pre-morbid functional status and pre-admission medications were not systematically documented. Missing data were handled by available-case analysis. In particular, admission lactate was missing in 34.0% of patients; to avoid resting the primary inference on an incomplete and potentially non-representative subset, the primary mortality model excluded lactate (n=265), and lactate was examined only in a secondary subset model (n=186) and in univariable and Kaplan-Meier analyses. Because lactate is preferentially measured in more acutely unwell patients, the secondary lactate estimate may be affected by selection and should be regarded as complementary. Because the secondary outcome was in-hospital mortality with discharge alive treated as censoring, discharge represented a competing event rather than non-informative censoring; a formal competing-risks framework was not applied, which may bias the time-to-event estimates. The modeling strategy (univariable screening at p<0.10 followed by backward elimination) carries a potential risk of overfitting despite bootstrap validation, and only in-hospital mortality was evaluated, without longer-term survival or functional recovery. As with all observational studies, residual confounding by unmeasured variables, including treatment-limitation decisions, cannot be excluded. Prospective, multicenter studies with formal frailty assessment, complete premorbid data, and extended follow-up are needed to validate and extend these observations.

## Conclusions

In this retrospective cohort of older adults admitted to a dedicated geriatric medical ICU, acute physiological derangement at presentation rather than chronological age or baseline comorbidity burden emerged as the dominant determinant of both invasive mechanical ventilation and in-hospital mortality. In overburdened units where formal frailty assessment is often unavailable, a physiology-first approach using routinely collected vital signs and basic laboratory tests enables rapid risk stratification, guides escalation of organ support, and informs timely goals-of-care discussions. Larger, multicenter prospective studies incorporating frailty and dynamic organ-failure trajectories are warranted to validate and refine this simple framework for broader application in aging populations across low-resource environments.
